# Associations of military divorce with mental, behavioral, and physical health outcomes

**DOI:** 10.1186/s12888-015-0517-7

**Published:** 2015-06-19

**Authors:** Lawrence Wang, Amber Seelig, Shelley MacDermid Wadsworth, Hope McMaster, John E. Alcaraz, Nancy F. Crum-Cianflone

**Affiliations:** 1 Deployment Health Research Department, Naval Health Research Center, San Diego, CA USA; 2 Purdue University, West Lafayette, IN USA; 3 San Diego State University, San Diego, CA USA

## Abstract

**Background:**

Divorce has been linked with poor physical and mental health outcomes among civilians. Given the unique stressors experienced by U.S. service members, including lengthy and/or multiple deployments, this study aimed to examine the associations of recent divorce on health and military outcomes among a cohort of U.S. service members.

**Methods:**

Millennium Cohort participants from the first enrollment panel, married at baseline (2001–2003), and married or divorced at follow-up (2004–2006), (*N* = 29,314). Those divorced were compared to those who remained married for mental, behavioral, physical health, and military outcomes using logistic regression models.

**Results:**

Compared to those who remained married, recently divorced participants were significantly more likely to screen positive for new-onset posttraumatic stress disorder, depression, smoking initiation, binge drinking, alcohol-related problems, and experience moderate weight gain. However, they were also more likely be in the highest 15^th^ percentile of physical functioning, and be able to deploy within the subsequent 3-year period after divorce.

**Conclusions:**

Recent divorce among military members was associated with adverse mental health outcomes and risky behaviors, but was also associated with higher odds of subsequent deployment. Attention should be given to those recently divorced regarding mental health and substance abuse treatment and prevention strategies.

## Background

The associations between divorce and mental and physical health disorders have been investigated in civilian populations [1–9], demonstrating that divorce is related to lower health-related quality of life [2], and greater disability and mortality [3, 4]. Recent research has shown that divorce is a risk factor for depression, posttraumatic stress disorder (PTSD), and elevated resting blood pressure, and is associated with other disorders such as seasonal affective disorder, social phobias, and bipolar disorder [5–7, 9]. Conversely, divorce has been linked to increased physical fitness in men [8]. Together, these findings present a clear picture of the physical and mental detriments associated with divorce among a civilian population.

Since the initiation of military operations in Iraq and Afghanistan after September 11^th^, the increased pace of military deployments has been shown to provide a protective effect against divorce [10] while at the same time, increasing the stress of military life and impacting the quality of marital relationships [11–13]. These differing results indicate that the relationship between deployment to Iraq and Afghanistan and divorce is not clear and additional studies are warranted [10, 14, 15]. Recent studies have demonstrated that infidelity and consideration of separation/divorce among military couples have increased during the recent conflicts [14, 15]. Further, service members returning from the war with PTSD or serious injuries, including traumatic brain injury, can strain the marital bond [12]. Overall, these data suggest the importance of exploring the potential effects of divorce on health and military outcomes among service members during an era of persistent conflicts, because these outcomes not only affect quality of life, but also military force readiness [16]. The purpose of this investigation was to utilize a large, representative military cohort, using a prospective design to study the extent to which participants experiencing a recent divorce reported subsequent poor health and military outcomes compared to those who remained married during the same time frame. We hypothesized that, among military, recent divorce is associated with worsened mental and behavioral health, improved physical capability and would have a negative association with deployment and military separation.

## Methods

### Population and data sources

The Millennium Cohort Study was designed to prospectively assess the short- and long-term health outcomes of service members, and was initiated in 2001 prior to September 11^th^. Individuals invited to participate in the Millennium Cohort Study were randomly selected from US military rosters with oversampling of selected sub-groups including women, Reserve and National Guard, and those who previously deployed (i.e., 1991 Gulf War or Bosnia, Kosovo, or Southwest Asia between 1998 and 2000) [17, 18]. Participants include active duty, Reserve, and National Guard personnel from all five service branches who voluntarily agreed to complete follow-up surveys approximately every 3 years. The first panel of participants were enrolled during 2001–2003 (*n* = 77,047), of whom 55,021 (71 %) completed a follow-up survey during 2004–2006. Additional details on Millennium Cohort Study methodology and response rates are described elsewhere [16, 17, 19, 20].

The current study evaluated participants in the first panel who were married at baseline and who were either married or divorced at the time of the follow-up survey (*n* = 34,500). Since military records were used to confirm marital status, participants who separated from military service before completing both surveys were excluded (*n* = 3419). In addition, participants missing demographic covariates (sex, birth year, education, race/ethnicity, *n* = 26), missing military covariates (combat deployment, prior deployment, service component, service branch, pay grade, military occupation *n* = 707), and with incomplete baseline physical, mental, or behavioral data (history of potential alcohol dependence, smoking status, body mass index, mental and physical functioning, *n* = 1034) were excluded. This study was approved by the San Diego State University and Naval Health Research Center Institutional Review Boards and written informed consent was obtained from all study participants.

Demographic and military data were obtained from electronic military personnel files, provided monthly by the Defense Manpower Data Center (DMDC). These files provided data on marital status, sex, birth date, race/ethnicity, education, service branch, service component, military pay grade, military occupation, dates of deployment, and date of military separation. Self-reported data collected on the Millennium Cohort Study questionnaires was used to assess all other variables in the analyses, including combat experiences.

### Outcome measures

Specific mental, physical, behavioral, and functional health outcomes were selected based on previous findings regarding their impact on force health and readiness [2–6, 21]. New-onset outcomes of interest were assessed only among those with no symptoms at baseline. Mental health outcomes included new-onset PTSD, new-onset depression, and anxiety/panic. The PTSD Checklist-Civilian Version (PCL-C), a 17-item symptom screening tool, was used to identify participants who screened positive for PTSD [22–25]. The PCL-C was scored using the specific criteria, where those who scored 50 or more on the PCL screened positive for PTSD symptoms. Participants screening positive for new-onset depression and anxiety/panic were identified using the PRIME-MD Patient Health Questionnaire (PHQ) [26–29]. The PHQ is a standardized tool to screen for mental health disorders based on the Diagnostic and Statistical Manual of Mental Disorders, 4th Edition.

Physical health outcomes included new-onset hypertension, new-onset diabetes, and weight change. Participants with diabetes and hypertension were identified using self-reported data on the Millennium Cohort questionnaire, where participants indicated a diagnosis from a health care professional. Weight change, determined using self-reported weight at baseline and follow-up, was calculated as the percent change from baseline to follow-up, and was categorized into five levels: extreme gain (>10 %), moderate gain (between 3 and 10 %), stable weight (within 3 % of baseline weight), moderate loss (between 3 and 10 %), and extreme loss (>10 %) [30].

Behavioral outcomes included new-onset alcohol-related problems, new-onset binge drinking, smoking initiation, and smoking recidivism. Alcohol-related problems were identified using the PHQ as making poor decisions regarding alcohol, such as driving under the influence or being hung over at work [27–29]. Binge drinking was defined as self-report of drinking five or more drinks on one occasion for men, and four or more drinks on one occasion for women [31]. Smoking initiation was defined as reporting current smoking at follow-up was assessed among baseline nonsmokers, and smoking recidivism, defined as report of current smoking at follow-up was assessed among baseline former smokers.

Functional health outcomes, assessed using the Mental Component Summary (MCS) score and Physical Component Summary (PCS) score, were included to portray a general assessment of mental and physical health status at follow-up. The MCS and PCS were calculated using the Medical Outcomes Study Short Form 36-item Survey for Veterans (SF-36 V), a measure designed to evaluate physical and mental functioning [32, 33]. Both MCS and PCS scores were categorized as one) lowest 15^th^ percentile, two) middle 70^th^ percentile, and three) highest 15^th^ percentile, where a higher score represents better functioning. The resulting groups correspond approximately to participants scoring more than one standard deviation below, within one standard deviation of, and more than one standard deviation above the mean.

Military-specific outcomes from the DMDC included deployment and separation from the military. Deployment (yes/no) was assessed during the 3-year period between the baseline and first follow-up survey in the Stayed Married group, and during the 3–year period following divorce for the Recently Divorced group. While the timing is different for each groups, the length of follow-up during which they had the opportunity to have the outcome (deployment) is the same. This method was chosen to ensure that the outcome occurred following the exposure, and that the time from exposure to outcome was the same. Separation from the military was assessed during the 3 years after the follow-up survey was completed for both groups, since all participants in these analyses remained in the military through their follow-up survey.

### Additional variables

Baseline characteristics were included in the analyses to adjust for factors as suggested by previous studies [19, 29, 34–49]. Variables included sex, birth year, education, race/ethnicity, military pay grade, service component, service branch, and military occupation. These variables were determined using DMDC records and were backfilled with self-reported data to reduce missing values. Baseline functional health was evaluated with the MCS and PCS using the categorization as described above. A prior history of potential alcohol dependence was assessed at baseline using the CAGE questionnaire (Cutting down, Annoyance by criticism, Guilty feeling, and Eye-opener); those that were categorized as having a history of potential alcohol dependence positively endorsed at least one of the CAGE questions [50, 51]. Baseline smoking status was determined using self-report, where current smokers were defined as those who indicated smoking at least 100 cigarettes in their lifetime and had either not tried to quit or had been unsuccessful at quitting. Baseline body mass index (BMI) was categorized as underweight (<18.5 kg/m^2^), normal range (18.5–24.9), overweight (25–29.9), or obese (≥30.0) [37].

Combat experience was defined as having personally witnessed at least one of the following events: death due to war, disaster or tragic event; physical abuse; dead or decomposing bodies; maimed soldiers or civilians; or prisoners of war or refugees. Combat was assessed at follow-up among deployers and was reported as having occurred during the same 3-year period as a deployment. Participants were categorized at baseline as deployed with combat experiences, deployed without combat experiences, and non-deployed. An additional deployment variable was included, describing deployment prior to 2001 and was assessed using military personnel records.

### Statistical analyses

Chi-square tests of association and unadjusted logistic regression models were used to compare characteristics between divorced and married service members. In multivariable analyses, separate logistic regression models were utilized to compare the odds of each outcome in relation to marital status while adjusting for relevant covariates. Polychotomous logistic regression was used to assess the tiered outcomes including weight change, MCS, and PCS. Multicollinearity was evaluated among all independent variables using a variance inflation factor of four. Final models were determined using a change in estimate approach, where variables were retained if significant (p < 0.05) or confounders (defined as a 10 % change in adjusted odds ratio). Sex and combat deployment were *a priori* maintained in the adjusted models. Based on this, the final models included sex, birth year, education, race/ethnicity, baseline smoking status, history of potential alcohol dependence, body mass index, baseline MCS and PCS, combat deployment, pay grade, service component, service branch, and occupation. Subanalyses were performed among service members divorced during follow-up, in which those who were divorced for > 2 years at their follow-up survey (reference group) were compared to those divorced for < 1 year, and those divorced for 1–2 years. Data management and statistical analyses were performed using SAS, version 9.3, statistical software (SAS Institute, Inc., Cary, North Carolina).

## Results

During the study period, 1545 (5.3 %) service members became divorced and 27,769 remained married. Service members who divorced were proportionally more likely at baseline to be female, younger, less educated, enlisted, active duty, under- or normal weight, and have lower mental and higher physical functioning compared to those who remained married (Table 1). All characteristics were significantly associated with the exposure at *p < 0.05*, except for service branch (*p = 0.74)*.

**Table 1:** Baseline demographic, military, and behavioral characteristics by marital status^a^

	Recently divorced		Stayed married		Total	
	*n* =1545		*n* = 27,769		*n* =29,314	
Baseline characteristics^b^	n	(%)	n	(%)	n	(%)
Sex						
Male	959	62.07	22800	82.11	23759	81.05
Female	586	37.93	4969	17.89	5555	18.95
Age						
Born before 1960	236	15.28	7581	27.30	7817	26.67
1960-1969	668	43.24	13195	47.52	13863	47.29
1970-1979	599	38.77	6767	24.37	7366	25.13
1980 and after	42	2.72	226	0.81	268	0.91
Education						
Some college or less	1212	78.45	18289	65.86	19501	66.52
Bachelor’s degree or higher	333	21.55	9480	34.14	9813	33.48
Race/Ethnicity						
White, non-Hispanic	1098	71.07	20171	72.64	21269	72.56
Black, non-Hispanic	214	13.85	2853	10.27	3067	10.46
Other	233	15.08	4745	17.09	4978	16.98
Combat deployment						
Not deployed	1030	66.67	19076	68.70	20106	68.59
Deployed, without combat	279	18.06	4345	15.65	4624	15.77
Deployed, with combat	236	15.28	4348	15.66	4584	15.64
Prior deployment						
Yes	445	28.80	9053	32.60	9498	32.40
No	1100	71.20	18716	67.40	19816	67.60
Military pay grade						
Enlisted	1263	81.75	18807	67.73	20070	68.47
Officer	282	18.25	8962	32.27	9244	31.53
Service component						
Active duty	873	56.50	14951	53.84	15824	53.98
Reserve/National Guard	672	43.50	12818	46.16	13490	46.02
Service branch						
Army	721	46.67	12940	46.60	13661	46.60
Navy/Coast Guard	228	14.76	4373	15.75	4601	15.70
Air Force	538	34.82	9432	33.97	9970	34.01
Marines	58	3.75	1024	3.69	1082	3.69
Military occupation						
Combat specialist	273	17.67	6402	23.05	6675	22.77
Health care	165	10.68	2997	10.79	3162	10.79
Other	1107	71.65	18370	66.15	19477	66.44
History of potential alcohol dependence^c^						
Yes	296	19.16	4617	16.63	4913	16.76
No	1249	80.84	23152	83.37	24401	83.24
Smoking status						
Current smoker	317	20.52	3958	14.25	4275	14.58
Other	1228	79.48	23811	85.75	25039	85.42
Body mass index^d^						
Underweight and normal weight	701	45.37	8670	31.22	9371	31.97
Overweight	717	46.41	15975	57.53	16692	56.94
Obese	127	8.22	3124	11.25	3251	11.09
Mental component summary score^e^						
Lowest 15^th^ percentile	296	19.16	2568	9.25	2864	9.77
Middle 70^th^ percentile	1043	67.51	19553	70.41	20596	70.26
Highest 15^th^ percentile	206	13.33	5648	20.34	5854	19.97
Physical component summary score^e^						
Lowest 15^th^ percentile	188	12.17	3604	12.98	3792	12.94
Middle 70^th^ percentile	1065	68.93	20675	74.45	21740	74.16
Highest 15^th^ percentile	292	18.90	3490	12.57	3782	12.90

^a^ Participants in this study completed a baseline survey in 2001–2003 and completed a follow-up survey in 2004–2006. Marital status assessed from baseline to first follow-up, where those who were “recently divorced” were married at their baseline survey and got divorced before their follow-up survey, those who “stayed married” were married throughout the study period

^b^ All characteristics were significantly associated with exposure at *p < 0.05*, except for service branch (*p = 0.74)*

^c^ Potential alcohol dependence is defined as at least 1 positive response to the CAGE questionnaire (Cutting down, Annoyance by criticism, Guilty feeling, and Eye-openers)

^d^ Body mass index is defined using Center for Disease Control and Prevention standards of Underweight (Less than 18.5 kg/m^2^), Normal range (18.5-24.99), Overweight (25–29.9), and Obese (≥30.0)

^e^ Mental component summary score and physical component summary score are obtained from the Short Form-36 Question Health Survey for Veterans (SF-36 V)

Table 2 displays the prevalence of each outcome by marital status. New-onset PTSD, depression, and anxiety were more prevalent among those recently divorced with 4.8 %, 3.0 %, and 3.0 % developing each of these conditions, respectively, between baseline and follow-up. Smoking initiation and recidivism, alcohol-related problems, and binge drinking were also more prevalent in divorced service members compared to those who remained married. Regarding physical health, weight gain was more prevalent in the divorced group, and married individuals were more likely to have a stable weight. Divorced service members were also more likely to be in the lowest 15^th^ percentile for mental functioning, and be in the highest 15^th^ percentile for physical functioning. Finally, divorced service members were proportionately more likely to deploy within 3 years following divorce, but had similar percentages of military separation. All outcomes were significantly associated with exposure at *p < 0.05*, except for new-onset diabetes (*p = 0.08)* and military separation (*p = 0.33)*.

**Table 2:** Prevalence of health and military outcomes by marital status^a^

	Recently divorced		Stayed married		Total	
	*N* = 1545		*N* = 27,769		*N* = 29,314	
Outcome^b^	n	(%)	n	(%)	n	(%)
Mental health						
New-onset posttraumatic stress disorder^b^	74	4.79	597	2.15	671	2.29
New-onset depression^c^	47	3.04	431	1.55	478	1.63
Anxiety/Panic^c^	47	3.04	476	1.72	523	1.78
Behavioral						
Smoking initiation	15	0.97	125	0.45	140	0.48
Smoking recidivism	53	3.43	685	2.47	738	2.52
New-onset alcohol-related problems^c^	62	4.01	727	2.62	789	2.69
New-onset binge drinking^d^	183	11.84	2846	10.25	3029	10.33
Physical health						
New-onset hypertension	84	5.44	1646	5.93	1730	5.90
New-onset diabetes	8	0.52	205	0.74	213	0.73
Weight change^e^						
Extreme weight loss	46	2.98	572	2.06	618	2.11
Moderate weight loss	169	10.94	3013	10.85	3182	10.85
Stable weight	572	37.02	12490	44.98	13062	44.56
Moderate weight gain	522	33.79	7949	28.63	8471	28.90
Extreme weight gain	120	7.77	1634	5.88	1754	5.98
Functional health						
Mental component summary score^f^						
Lowest 15^th^ percentile	242	15.66	2761	9.94	3003	10.24
Middle 70^th^ percentile	1061	68.67	19563	70.45	20624	70.36
Highest 15^th^ percentile	221	14.30	4999	18.00	5220	17.81
Physical component summary score^f^						
Lowest 15^th^ percentile	211	13.66	3633	13.08	3884	13.11
Middle 70^th^ percentile	1031	66.73	19928	71.76	20959	71.50
Highest 15^th^ percentile	282	18.25	3762	13.55	4044	13.80
Military related outcomes						
Deployment^g^	541	38.26	8693	31.30	9234	31.64
Military separation^h^	205	13.27	3929	14.15	4134	14.10

^a^ Marital status assessed from baseline to first follow-up, where those who were “recently divorced” were married at their baseline survey and got divorced before their follow-up survey, those who “stayed married” were married throughout the study period. The reference category is “stayed married”

^b^ All outcomes were significantly associated with exposure at *p < 0.05*, except for new-onset diabetes (*p = 0.08)* and military separation *(p = 0.33)*

^c^ Posttraumatic stress disorder is defined using the PTSD checklist-civilian version, specific criteria with a cutoff of 50

^d^ Assessed using the Patient Health Questionnaire

^e^ Binge drinking is defined as reporting 5 or more drinks per occasion for men or 4 or more drinks per occasion for women on at least 1 day during the past year

^f^ Extreme weight loss (≥10 % loss), moderate weight loss (between 3 % and 10 % loss), stable weight (within ± 3 %), moderate weight gain (between 3 % and 10 % gain), and extreme weight gain (≥10 % gain)

^g^ Mental component summary score and physical component summary score are obtained from the short form 36 question health survey for veterans (SF-36 V)

^h^ Deployment was assessed from baseline until the follow-up for those in the “stayed married” and in the 3 years following divorce for the “recently divorced” group

^i^ Separation was assessed after the date of the completion of the follow-up survey

In the multivariable models (Table 3), recently divorced service members were more likely to screen positive for new-onset PTSD [Adjusted Odds Ratio (AOR) 1.77; 95 % Confidence Interval (CI): 1.36, 2.30] and new-onset depression (AOR: 1.40; 95 % CI: 1.02, 1.94) than those individuals who stayed married. Models focusing on behavioral health outcomes showed that those who were recently divorced were more likely to initiate smoking (AOR: 2.04; 95 % CI: 1.18, 3.54), develop alcohol-related problems (AOR: 1.52; 95 % CI: 1.15, 2.00), and initiate binge drinking (AOR: 1.51; 95 % CI: 1.26, 1.81) than those who remained married. Regarding physical health outcomes, those who were recently divorced were more likely to experience moderate (between 3 % and 10 %) weight gain (AOR: 1.15; 95 % CI: 1.02, 1.31) than those who remained married. Finally, service members who were recently divorced were more likely to be in the highest 15^th^ percentile of physical health functioning based on the PCS score (AOR: 1.26; 95 % CI: 1.10, 1.46). After adjusting for other covariates in the reduced model, analyses examining deployment and military separation showed that those divorced were more likely to deploy (AOR:1.47; 95 % CI: 1.31, 1.66) than those people who stayed married. Experiencing a recent divorce was not found to be significantly associated with separation from the military.

**Table 3:** Adjusted^a^ odds of health and military outcomes comparing recently divorced military personnel with those who stayed married^b^

	Divorced vs. married	
Outcome^c^	AOR	(95 % CI)
Mental health		
New-onset posttraumatic stress disorder^d^	1.77^k^	(1.36, 2.30)
New-onset depression^e^	1.40^k^	(1.02, 1.94)
Anxiety/panic^e^	1.24	(0.90, 1.71)
Behavioral		
Smoking initiation	2.04^k^	(1.18, 3.54)
Smoking recidivism	1.25	(0.93, 1.68)
New-onset alcohol-related problems^e^	1.52^k^	(1.15, 2.00)
New-onset binge drinking^f^	1.51^k^	(1.26, 1.81)
Physical health		
New-onset hypertension	1.13	(0.89, 1.42)
New-onset diabetes	0.82	(0.40, 1.69)
Weight change^g^		
Extreme weight loss	1.31	(0.94, 1.80)
Moderate weight loss	1.08	(0.90, 1.29)
Stable weight	1.00	-
Moderate weight gain	1.15^k^	(1.02, 1.31)
Extreme weight gain	0.90	(0.73, 1.12)
Functional health		
Mental component summary score^h^		
Lowest 1 ^th^ percentile	1.09	(0.93, 1.28)
Middle 70^th^ percentile	1.00	-
Highest 15^th^ percentile	1.02	(0.87, 1.19)
Physical component summary score^h^		
Lowest 15^th^ percentile	1.01	(0.86, 1.20)
Middle 70^th^ percentile	1.00	-
Highest 15^th^ percentile	1.26^k^	(1.10, 1.46)
Military related outcomes		
Deployment^i^	1.47^k^	(1.31, 1.66)
Military separation^j^	0.95	(0.80, 1.11)

^a^ Full models were adjusted for all variables: sex, birth year, education, race/ethnicity, baseline smoking status, history of potential alcohol dependence, body mass index, baseline MCS and PCS, combat deployment, pay grade, service component, service branch, and occupation. Reduction of models utilized backwards stepwise regression

^b^ Marital status assessed from baseline to first follow-up, where those who were “recently divorced” were married at their baseline survey and got divorced before their follow-up survey, those who “stayed married” were married throughout the study period. The reference category is “stayed married”

^c^ In outcomes without a reference indication, the reference category is “No”

^d^ Posttraumatic stress disorder is defined using the PTSD checklist-civilian version, specific criteria with a cutoff of 50. ^e^ Assessed using the Patient Health Questionnaire

^f^ Binge drinking is defined as reporting 5 or more drinks per occasion for men or 4 or more drinks per occasion for women on at least 1 day during the past year

^g^ Extreme weight loss (≥10 % loss), moderate weight loss (between 3 % and 10 % loss), stable weight (within ± 3 %), moderate weight gain (between 3 % and 10 % gain), and extreme weight gain (≥10 % gain)

^h^ Mental component summary score and physical component summary score are obtained from the Short Form-36 Question Health Survey for Veterans (SF-36 V)

^i^ Deployment was assessed from baseline until the follow-up for those in the “stayed married” and assessed until 3 years after divorce event for the “recently divorced” population

^j^ Separation was assessed after the date of the completion of the follow-up survey

^k^ Adjusted Odds Ratio was found to be statistically significant

Among divorced service members, we examined the association of the timing between the divorce and the assessment of health and military outcomes. We compared those who completed their survey within 1 year after their divorce date with those who had between 2 and 3 years between their divorce and completion of survey. The latter served as the reference group. In the adjusted model, those who completed the survey within a year of their divorce date were significantly more likely to screen positive for new-onset PTSD (AOR: 3.27; 95 % CI: 1.56, 6.86), have alcohol-related problems (AOR: 2.24; 95 % CI: 1.11, 4.53), and binge drinking (AOR: 1.90; 95 % CI: 1.18, 3.06) compared to those who completed the survey between 2 and 3 years after the divorce date (Table 4). They were also less likely to report extreme weight gain (AOR: 0.41; 95 % CI: 0.23, 0.71). We also compared those who had completed their survey between 1 and 2 years after their divorce. The reference group, those with between 2 and 3 years between their divorce and completion of survey, remained the same. In the adjusted model, those who had between 1 and 2 years since divorce were less likely to report binge drinking (AOR: 0.53; 95 % CI: 0.31, 0.90) compared to those who completed the survey between 2 and 3 years after their divorce.

**Table 4:** Adjusted^a^ odds of health and military outcomes by timing of divorce^b^

	<1 year since divorce		1-2 years since divorce	
Outcome^c^	AOR	(95 % CI)	AOR	(95 % CI)
Mental health				
New-onset posttraumatic stress disorder^d^	3.27^j^	(1.56, 6.86)	1.79	(0.82, 3.92)
New-onset depression^e^	1.16	(0.53, 2.53)	1.19	(0.54, 2.60)
Behavioral				
Smoking initiation	0.83	(0.25, 2.78)	0.53	(0.14, 2.04)
New-onset alcohol-related problems^e^	2.24^j^	(1.11, 4.53)	1.07	(0.50, 2.29)
New-onset binge drinking^f^	1.90^j^	(1.18, 3.06)	0.53^j^	(0.31, 0.90)
Physical health				
Weight change^g^				
Extreme weight loss	1.82	(0.80, 4.14)	0.93	(0.37, 2.31)
Moderate weight loss	1.54	(0.97, 2.43)	0.93	(0.57, 1.51)
Stable weight	1.00	-	1.00	-
Moderate weight gain	0.85	(0.62, 1.16)	0.93	(0.69, 1.27)
Extreme weight gain	0.41^j^	(0.23, 0.71)	0.87	(0.54, 1.41)
Functional health				
Physical component summary score^h^				
Lowest 15^th^ percentile	0.86	(0.57, 1.29)	0.90	(0.60, 1.34)
Middle 70^th^ percentile	1.00	-	1.00	-
Highest 15^th^ percentile	1.32	(0.92, 1.88)	0.96	(0.67, 1.38)
Military related outcomes				
Deployment^i^	1.17	(0.87, 1.56)	1.05	(0.79, 1.40)

^a^ Full models were adjusted for all variables: sex, birth year, education, race/ethnicity, baseline smoking status, history of potential alcohol dependence, body mass index, baseline MCS and PCS, combat deployment, pay grade, service component, service branch, occupation

^b^ Marital status assessed from baseline to first follow-up, where those who were “recently divorced” were married at their baseline survey and got divorced before their follow-up survey. The reference category is > 2 years since divorce

^c^ In outcomes without a reference indication, the reference category is “No”

^d^ Posttraumatic stress disorder is defined using the PTSD checklist-civilian version, specific criteria with a cutoff of 50

^e^ Assessed using the Patient Health Questionnaire

^f^ Binge drinking is defined as reporting five or more drinks per occasion for men or four or more drinks per occasion for women on at least 1 day during the past year

^g^ Extreme weight loss (≥10 % loss), moderate weight loss (between 3 % and 10 % loss), stable weight (within ± 3 %), moderate weight gain (between 3 % and 10 % gain), and extreme weight gain (≥10 % gain)

^h^ Physical component summary score are obtained from the Short Form-36 Question Health Survey for Veterans (SF-36 V)

^i^ Deployment was assessed from baseline until the follow-up for those in the “stayed married” and assessed until 3 years after divorce event for the “recently divorced” population

^j^ Adjusted Odds Ratio was found to be statistically significant

## Discussion

This prospective study evaluated the associations of divorce on a comprehensive set of mental, behavioral, physical, and functional health outcomes in a large military cohort during the Iraq and Afghanistan wars. Data on the impact of divorce in a military setting are especially important given the increasing rates of marital distress associated with the recent conflicts [10, 12, 14]. After controlling for baseline sociodemographics and health status, this study showed significant associations between divorce and new-onset PTSD, depression, smoking initiation and recidivism, alcohol-related problems, binge drinking, and moderate weight gain. Study results suggest that special attention should be given to recently divorced individuals regarding mental health and risky health-related behaviors treatment and prevention strategies.

Studies of the consequences of divorce in the general population have revealed a variety of consequences for both adults and children. Although health implications for adults are in part due to selection effects, the bulk of the evidence attributes negative outcomes to the effects of divorce rather than selection [52]. Divorce can result in both acute and chronic stress for adult partners, with significant emotional upheaval that can include conflict, resentment, anger, and sadness [52]. In the military, divorce may mean loss of access to on-base housing or other changes in living arrangements, loss of medical benefits, and reduced family income as well as loss of spousal support and assistance. Although both short- and long-term consequences can occur, declines in well-being are temporary for most individuals [53].

This study found significant associations between divorce and the development of new-onset PTSD. Previous research regarding the relationship of divorce and PTSD has found bidirectional relationships. PTSD has been linked to elevated levels of hostile behavior, decreased capacity for intimacy, marital distress, and domestic violence [54, 55], all which may increase the likelihood of divorce. In a study examining male Vietnam veterans, those with PTSD had more problems in their relationships, more difficulties with intimacy, and had taken more steps toward separation and divorce than veterans not screening positive for PTSD [56]. Life events such as divorce may also generate symptoms of post-traumatic stress similar to other types of trauma such as accidents or abuse [57]. Our study results showed a relationship between divorce and screening positive for PTSD within a 3-year period when compared to those who remained married. This suggests that the mental health effects of divorce occur over a relatively short time period, information that may be helpful for providers caring for recently divorced individuals.

Similarly, our study found a significant association between divorce and depression, which is consistent with results from previous research, including a civilian study that found an association as early as 4-years [58], though our results show a shorter time frame of ≤3 years. Of note, the association between divorce and depression was present when comparing recently divorced service members to those who remained married, but depression was similar among recent divorcees and those who were single. Understanding the temporal proximity of the association between a recent divorce and mental health issues may help enable faster detection and management of these conditions.

Alcohol-related problems are detrimental to overall occupational performance especially during military operations. The present study showed a statistically significant association between divorce and alcohol-related problems and binge drinking. Previous studies have shown that drinking problems increase the risk of divorce [9, 59]. Our results, after controlling for sociodemographic factors and potential alcohol dependence at baseline, support an association between divorce and alcohol use. A longer follow-up period with evaluation of the temporal sequence could further elucidate the link between divorce and alcohol misuse. Our results also indicated that recent divorcees were significantly more likely to initiate smoking. These data support the hypothesis that marital support may be advantageous in reducing risky health-related behaviors [60].

The results of our study indicated a relationship between a recent divorce and improved physical function, and counterintuitively, also moderate weight gain. Given that there was no association with more extreme weight changes, and previous observational research has reported that divorce is associated with increased physical fitness in men [8], we hypothesize that divorce may be linked to increased motivation for physical fitness or the use of exercise to mitigate stress, and that the moderate gain is due to an increase in muscle mass. It is also possible that the moderate weight gain and improved physical fitness is due to the combination of an increase in exercise and alcohol consumption. The exact etiology is impossible to determine using this data, but our incongruent results suggest additional study is needed.

We investigated the hypothesis that a recent divorce may have a negative link with subsequent deployments. However, we found that those who were recently divorced were more likely to deploy within a 3-year period than those who remained married, suggesting that divorce did not adversely impact deployment eligibility. Recently divorced service members may be more likely to volunteer to deploy as a means to escape emotional hardship, although this study did not collect data on reasons for deployment. The study did not find an association between divorce and separation from the military, suggesting that marital status change may not directly affect the military careers of service members in the short term, but further studies are needed.

The time dependent analysis showed that service members who were recently divorced (<1 year) were more likely to report new-onset PTSD, alcohol-related problems, and binge drinking, compared to those who were divorced for >2 years. These findings suggest that mental and behavioral health outcomes are more likely to be reported within a shorter time frame, which is consistent with a previous study that found that length of time from marital separation was the most powerful factor in emotional resolution [61]. Nonetheless, even short-term health and behavioral conditions demonstrated in this study may interfere with both relationships and work performance, and may influence future behaviors. It is also possible that the symptoms detected by screening instruments in this study were a result of traumatic experiences and processes of grief associated with divorce, but do not represent chronic mental or behavioral health conditions.

Our study has notable limitations. Study outcomes were self-reported and were not validated by medical record review; however, the use of validated surveys to detect mental and behavioral health conditions in a military sample has several advantages because these conditions are often underdiagnosed due to stigma [62]. Although previous studies on the Millennium Cohort found it to be representative, responders were proportionally more likely to be women, educated, and white non-Hispanic [16, 63]. Because our follow-up period was 3 years and certain outcomes may develop later, we may have had inadequate time to detect these outcomes. Conversely, the effects of outcomes may not be long-lasting and may have been underestimated. Analyses were only able to assess temporal proximity as opposed to temporal sequence, and thus we were unable to establish causality. PTSD in particular is a complex affliction that is underreported and may also take variable lengths of time to develop, which may cloud possible associations with divorce. Additionally, the number of legally married study participants is also likely to be conservative in relation to real population of military couples, which includes common law and same-sex marriages. Finally, we did not have data on length of marriage or presence of children, which have been shown to affect the marital relationship [64]. A measure of marital satisfaction at baseline would also have been useful to further elucidate the relationship between divorce and health outcomes.

There are also significant strengths. Few data exist regarding the effects of divorce on physical and mental health outcomes directly related to military readiness and capability. The Millennium Cohort Study represents all branches of the US military and unlike many previous studies, our prospective study was able to assess new-onset outcomes. The large sample allowed adequate power for adjustment of many covariates, resulting in more robust comparisons and the ability to detect small differences within subgroups of the study population.

## Conclusion

Recent divorce was associated with screening positive for new-onset PTSD, depression, moderate weight gain, tobacco and alcohol use. Despite these findings, recent divorcees were more likely to be physically active and deploy compared to those who remained married. These data suggest that recently divorced service members should be screened for mental health and behavioral risk factors to improve their overall health and readiness. Given the negative health repercussions of divorce, future studies should identify factors associated with divorce in the military setting to reduce the development of these outcomes.

This work represents report 13–40, supported by the Department of Defense, under Work Unit No. 60002. The views expressed in this article are those of the authors and do not reflect the official policy or position of the Department of the Navy, Department of the Army, Department of the Air Force, Department of Defense, Department of Veterans Affairs, or the US Government. Approved for public release; distribution is unlimited. This research was conducted in compliance with all applicable federal regulations governing the protection of human subjects (Protocol NHRC.2000.0007).
